# Chromosomal radiosensitivity in breast cancer patients with a known or putative genetic predisposition

**DOI:** 10.1038/sj.bjc.6601188

**Published:** 2003-08-26

**Authors:** C B Seymour, C Mothersill, M Moriarty

**Affiliations:** 1 Saint Lukes Institute of Cancer Research, Oakland, Highfield Road, Rathgar, Dublin 6, Ireland; 2 Radiation and Environmental Science Centre, Dublin Institute of Technology, Dublin 8, Ireland

**Sir**,

The paper by Baeyens *et al* (2002) is interesting and we would like to contribute to the discussion. The authors suggest that the lack of correlation observed between G2 and G0 chromosomal radiosensitivity, also observed by Scott *et al* (1994), could be attributable to different DNA damage processing mechanisms operating in the G0 and G2 phase of the cell cycle.

It is also possible that different radiation pathways are involved. The G2 assay was performed following a dose of 0.4 Gy. We and others have demonstrated that this is the dose where maximal indirect nontargeted radiation cell killing (the bystander effect) occurs (Seymour and Mothersill, 2000). In our experience this effect plateaus at this dose, and a dose-dependent cell killing then follows. G0 is a specific stage of the cell cycle, which in the past has been demonstrated to allow PLD repair, that is potentially lethal damage that would in fact be lethal were the cell not in G0 (Hall, 2000). The observed result with 3.5 Gy HDR (45% radiosensitive) compared with the G2 results for 0.4 Gy (43% radiosensitive) could be interpreted as suggesting that radiosensitivity is independent of direct radiation damage, and is rather a function of indirect effects. The lack of correlation between the end points (G2-HDR MN *r*=0.04 and G2-LDR MN *r*=0.05) could then be attributable to differences in bystander expression during different stages of the cell cycle. The effect of the low dose rate in increasing the radiosensitivity is difficult to explain in terms of conventional radiobiology, but an inverse dose rate effect is well documented in the literature (Geard *et al*, 1994). The role of genetic predisposition to cancer in determining G2 radiosensitivity is also interesting. Our group has shown genetic variation of bystander-induced cell death (Mothersill *et al*, 2001), but our interpretation has been to link cancer proneness with lack of cell death following exposure of unirradiated cells to the medium harvested from irradiated cells. Clearly this is an interesting area awaiting clarification.
